# Combined application of CRISPR-Cas and stem cells for clinical and basic research

**DOI:** 10.1186/s13619-020-00062-4

**Published:** 2020-10-09

**Authors:** Meng Yan, Jinsong Li

**Affiliations:** 1 State Key Laboratory of Cell Biology, Shanghai Key Laboratory of Molecular Andrology, Shanghai Institute of Biochemistry and Cell Biology, Center for Excellence in Molecular Cell Science, Chinese Academy of Sciences; University of Chinese Academy of Sciences, Shanghai, 200031 China; 2 School of Life Science and Technology, ShanghaiTech University, 100 Haike Road, Shanghai, 201210 China; 3 School of Life Science, Hangzhou Institute for Advanced Study, University of Chinese Academy of Sciences, Hangzhou, 310024 China

Genetic disorders pose as a significant threat to the well-being of human life in this century, as more and more diseases identified are related to mutations presented in patients’ genome. Since the revolution of NGS (Next Generation Sequencing) technology and family tree analytics, mutations causing disease can be located relatively easily, thus granting doctors the time needed in searching for a treatment. Till 7 years ago, it’s not been easy for scientists to correct genomic mutations, even there were tools with promising implementations. Nevertheless, all have been changed with the debut of CRISPR (Clustered Regularly Interspaced Short Palindromic Repeats) technology, one that possesses the real potential to develop a treatment or even a cure for genetic mutation related diseases due to its simplicity and high-efficiency. In 2012, the mechanism of CRISPR-Cas9 in defending foreign phage used by bacterium has been just revealed. Now, just in 8 years, a plethora of CRISPR-related tools have been developed to act on multiple situations requiring genetic or epigenetic manipulations, such as CRISPR-Cas-based base editors, transposases/recombinases, prime editors, transcriptional regulators, RNA editors, epigenetic modulators and DNA/RNA detectors (Anzalone et al. 2020). Especially in the on-going COVID-19 pandemic, a faster and easier assay based on CRISPR-Cas12 has been developed for detection of the virus (Broughton et al. 2020). The exponential increase of CRISPR studies every year pushes CRISPR technology on a fast wheel, even clinical human trials have been reported recently (Maeder et al. 2019), and we believe that many more cases are underway to test the limits and viability of CRISPR technology.

Concerns over CRISPR technology have been risen alongside its ever-forwarding steps, focusing on safety, efficiency and ethical aspects of the new star (Doudna 2020). First, since its beginning, the off-target phenomena of CRISPR technology has been a major concern, because it poses a great threat to genomic integrity of non-target sites, which may cause unwanted side effects. Improvements regarding to this problem have been proposed over the years, for instance, computer-assisted protein design of Cas9 and machine learning algorithm-assisted target sequence screen or efficiency prediction, which can lower off-target events, but comes at the cost of decreasing efficiency. Second, there are limited target sites due to requirement of PAM sequence for DNA cleavage, such as NGG for Cas9. On target genome, the location of PAM sequence permits only a small portion of genome sites to be targeted; it is even more crucial for base editors, which only function in a relatively small window. Endeavors of many scientists have extended the targeting scope of CRISPR, making it possible to target more sites. However, the drawback of these studies is the decreased robustness of Cas9 activity and increased opportunity of off-target events, which is preventing itself from practical applications in treating human genetic diseases. Third, the challenge of delivering CRISPR system to the target tissue remains (van Haasteren et al. 2020). Delivery of the CRISPR system could be in the form of DNA, RNA or protein-RNA complex through viral vectors, nanoparticles, direct cell injection, or electroporation. Each method has its own advantages and limitations in aspects of genome integration, efficacy, tissue-specificity, and off-target activity. Last, the safety and ethical issues of the CRISPR applications need re-evaluations along with the discovery of more details about its capabilities. Apart from off-target risks, the immunogenicity of the CRISPR system and pre-existing CRISPR component antibody could offset all its benefits, and that would dramatically affect its application in disease treatment. So, large scale human trials of the CRISPR system are necessary before going routine in clinical applications, and trials must follow the law and strict local clinical requirements to avoid non-permitted abuse of CRISPR system.

To disperse all the concerns and make the CRISPR system application a clinical solution, there is still a long way to go, and we cannot afford to take any shortcuts. However, in the near future, there is one possible workaround that involves stem cell technology (Kimbrel and Lanza 2020). Stem cells, unlike the newbie CRISPR system, are used clinically for decades, and provided hundreds and thousands of successful life-saving examples. Those cells are able to self-renew and differentiate into functional somatic cells. Importantly, some stem cells can be long-term maintained in vitro without losing their stem cell capability. One significant advantage of combined usage of stem cells and the CRISRP system is that fully functional HDR (Homology-Directed Repair) machinery is generally active only in dividing cells, which is crucial for CRISPR related gene repair and knock-in system. Unwanted CRISPR off-target mutations after genetic manipulations in cultured stem cells can also be easily detected through NGS, and thus minimize the side effects of the CRISPR system for clinical applications. While ethnical debates around human embryonic stem cells limit its development, the derivation of iPSCs (induced Pluripotent Stem Cells) through exogenously expressing Yamanaka factors in differentiated somatic cells has largely migrated the debates. Patient-originated iPSCs with pluripotency similar to embryonic stem cells have the potential to differentiate into functional somatic cells in vitro for transplantation therapies, thus providing patient-specific therapeutic solutions. Brilliant as it is, but those kinds of properties that iPSCs possess may come with unexpected genetic and epigenetic defects. In a lot of cases, its tumorigenicity in vivo would put out all the possible clinical usages. Nonetheless, iPSCs are still suitable for patient-tailored drug screen and testing. With the powerful CRISPR system, scientists are getting more details about the tumorigenicity properties of iPSCs, in hoping to lower their tumor turnover rates. In addition, studies on cancer-genic genes in iPSCs-cancer cell transition, might turn out valuable for cancer treatment (Yin et al. 2019).

Naturally-occurring stem cells in adult human tissues are another kinds of stem cells that only have restricted potency (Blau and Daley 2019). Those adult stem cells maintain unipotency or multipotency, and can only differentiate into specialized cell populations, which are less cancer-prone and makes them safer for therapeutic purposes. HSCs (Hematopoietic Stem Cells), which have been used in bone marrow transplant to treat patients with hematological cancers, serve as an excellent target for the CRISPR system to achieve its full potential. A recent study has attempted to incorporate the CRISPR system to knockout *CCR5* in HSPCs (Hematopoietic Stem and Progenitor Cells) of a patient with HIV infection and acute lymphoblastic leukemia (Xu et al. 2019). After transplantation of *CCR5* mutated HSPCs, the patient exhibited normal physiological reactions with no adverse effects; and more importantly, those CRISPR edited HSPCs could well engrafted into patient’s system, although at a low rate. These results indicate that the CRISPR system is really a promising tool for genetic manipulations in adult human stem cells. Nevertheless, the therapeutic application of the CRISPR system is still in its infancy. More and more trials are required to promote its clinical efficacy and safety in the future.

Major questions of adult stem cells are whether targeting tissues have their stem cells, and whether these tissue-specific stem cells can be isolated and stably maintained in vitro for a sufficient period of time. Those are the essential steps to take before their clinical applications. The key to address these questions depends on the basic studies of stem cells. With the fast-developing biological technologies, such as single-cell sequencing and organoid formation, two recent studies have shown the existence of progenitor cells in pancreatic islet and prostate, and these cells can be long-term expanded in vitro (Guo et al. 2020*;* Wang et al. 2020). Taken together, CRISPR technology’s clinical applications based on stem cells would mostly remain in experimental stages now. Basic and pre-clinical experiments using stem cells would accelerate the discovery and development of cell-based targeted gene therapies.
